# Synthesis, Characterization and Antimicrobial Activity of New 1,2,3-Selenadiazoles

**DOI:** 10.3390/molecules13112740

**Published:** 2008-11-03

**Authors:** Mousa Al-Smadi, Fouad Al-Momani

**Affiliations:** 1 Department of Applied Chemical Sciences, Jordan University of Science and Technology, P.O.Box 3030, Irbid 22110, Jordan; 2 Department of Applied Biological Sciences, Jordan University of Science and Technology, P.O.Box 3030, Irbid 22110, Jordan

**Keywords:** Ketones, tosylhydrazones, 1,2,3-selenadiazoles, epoxide, antimicrobial activity

## Abstract

The commercially available aromatic polyketones **1a-d** were utilized for the synthesis of the multi-arm1,2,3-selenadiazole derivatives **3a-d**. The preparation starts with the reaction between compounds **1a-d** and *p*-toluenesulfonyl hydrazide to give the corresponding tosylhydrazones **2a-d**. Subsequent reaction with selenium dioxide leads to regiospecific ring closure of the tosylhydrazones to give the target multi-arm 1,2,3-selenadiazole derivatives in high yield. A 1,2,3-selenadiazole derivative **3e** containing an epoxide ring was also prepared. The structures of all the synthesized compounds were confirmed on the basis of spectral and analytical data. The compounds were screened *in vitro* for their antimicrobial activity against various pathogenic bacterial and *Candida* strains obtained from King Abdullah Hospital in Irbid -Jordan. Compounds **3a**, **3c** and **3e** were found to be highly active against all the selected pathogens. Compound **3e **showed an inhibition zone of 13 mm against the highly resistant *P. aruginosa*.

## Introduction

Selenium containing heterocyclic compounds are of interest due to their biological and synthetic applications. 1,2,3-Selenadiazoles and derivatives are well known and have attracted attention as versatile synthetic intermediates [1,2]. Many substituted 1,2,3-selenadiazoles and derivatives have been prepared to-date and some of them show high antibacterial activity [3,4,5,6]. The antifungal activity of other substituted 1,2,3-selenadiazoles has also been determined [6,7,8]. It has been found that the introduction of a 1,2,3-selenadiazole ring to molecules of known biological activity compounds changes their activities and in some cases leads to an increase in their biological activity [9]. Other heterocyclic compounds containing five membered rings like triazole, oxazole, pyrazoline, pyrazole and thiazole have been found to be biologically active substances [3, 11,12]. These ring systems are present in numerous antiparasitic, fungicidal, antihelminitic and antiinflammatory drugs.

β-Lactam antibiotics derivatized with a 1,2,3-thiadiazole-5-mercapto moiety have been found to be active against Gram-negative bacteria such as *Pseudomonas aeruginosa*. 4-Methyl-1,2,3-selena-diazole-5-carboxamides have been described to inhibit tumor cell colony formation [13,14]. In the area of antibacterial therapeutics, resistance to currently available drugs is progressively limiting their utility in treating bacterial infections. This problem can be solved by discovering novel pharmaceutical drugs that inhibit novel targets. Advances in molecular microbiology and genomics have led to the identification of numerous bacterial genes that are encoding for novel proteins, that could potentially serve as novel targets for antibacterial compounds. Regulatory proteins such as the two-component histidine kinases, involved in bacterial signal transduction, have recently gained considerable attention as one such class of potential targets [15].

As a continuation of our work on the synthesis of heterocyclic compounds containing 1,2,3-thiadiazoles and 1,2,3-selenadiazoles [16,17,18], we have set out to synthesize a new group of heterocyclic compounds containing 1,2,3-selenadiazole derivatives from the corresponding ketones **1a-d**, respectively, hoping to obtain biologically active compounds with potential use in the manufacture of pharmaceutical drugs. In this paper we present new data on the antimicrobial activities of heterocyclic compounds containing 1,2,3-selenadiazole rings and the corresponding tosylhydrazones.

## Results and Discussion

**Scheme 1:**
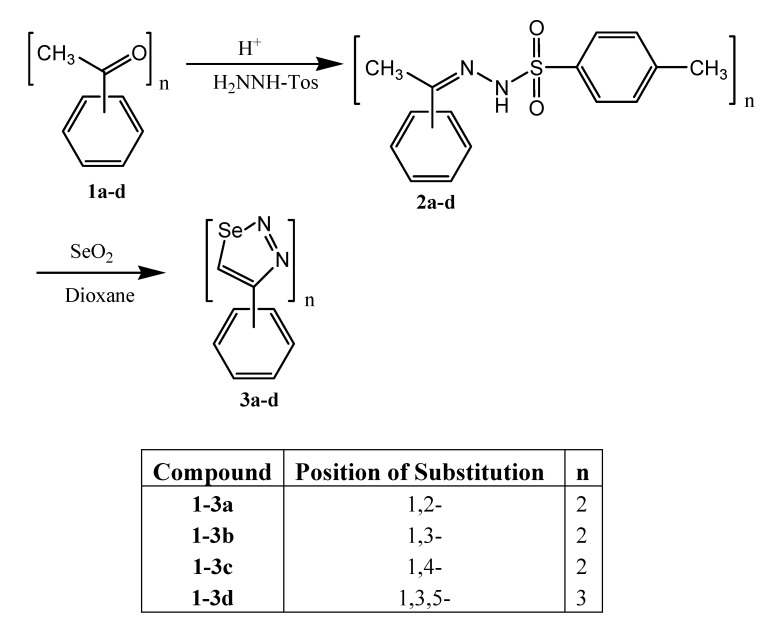


**Scheme 2:**
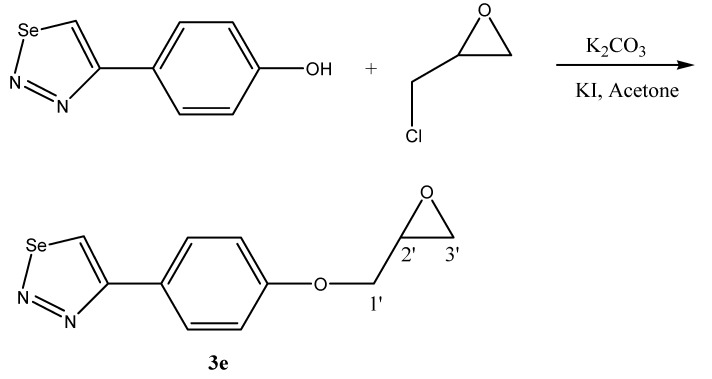


The target 1,2,3-selenadiazole derivatives **3a-d** were prepared from the corresponding tosyl-hydrazones **2a-d** as shown in Scheme 1. The synthetic procedure started from the commercially available polyketones **1a-d**. They were transformed into the target heterocyclic compounds by the reaction of the tosylhydrazones with selenium dioxide, as previously described by Lalezari *et. al.* [19,20]. Compound **3e** was prepared by reaction of 4-(1,2,3-selenadiazole-4-yl)phenol [16] and epichlorohydrin, as shown in Scheme 2.

The activity of these heterocyclic compounds and their tosylhydrazone precursors was tested against some human pathogenic microbes including Gram-positive (*Staphylococcus aureus*), Gram-negative (*Escherichia coli*, local resistant *Pseudomonas aeruginosa* and a reference *Pseudomonas* *aeruginosa* ATCC 27853) and *Candida albicans* by two methods, namely by the hole diffusion (see Table 1) and by the paper disk method (see Table 2).

From the results obtained from the two methods (Table 1 and Table 2), it was found that some of the tested heterocyclic compounds were highly active even at low concentrations (ie., at 0.005 g/mL or less). Both methods indicated that the sensitivity of the highly resistance *Pseudomonas*. *aeruginosa* and the sensitivity of a local, highly resistant clinical isolate, collected from King Abdullah Hospital at Jordan University of Science and Technology, to some of these heterocyclic compounds is high.

The extracts of compounds **3a**, **3c** and **3e** in dimethyl sulfoxide (DMSO) are active against all the tested pathogens, with a (8-19 mm) inhibition zone diameter, when using the hole diffusion technique, as indicated in Table 1. *Pseudomonas. aeruginosa*, a local isolate highly resistant to all domestic antibiotics (amikacin, carbenicillin, cefoperazone, gentamicin, tobramycin), used in the clinical labs of King Abdullah Hospital was found to be more sensitive to compound **3e**. This isolate was also sensitive to compounds **3a** and **3c** (at 0.01 g/mL) and to **3e** (at 0.005 g/mL). The solvent showed no activity against any of the tested pathogens.

**Table 1:** Sensitivity of human pathogenic microbes to the new synthetic heterocyclic compounds using the hole method.

Compound	Conc. (g/mL)	Pathogens				
		A^a^	B	C	D	E
**Tobramycin**	0.010	15^b ^	13	22	-	14
	0.005	13	11	17	-	8
						
**2a**	0.010	11	12	12	-	9
	0.005	8	10	8	-	-
**2b**	0.010	13	13	14	-	8
	0.005	8	8	9	-	-
**2c**	0.010	-	8	11	-	-
	0.005	-	-	8	-	-
**2d**	0.010	-	9	10	-	8
	0.005	-	-	8	-	-
**3a**	0.010	8	12	10	8	14
	0.005	-	9	8	-	10
**3b**	0.010	9	11	11	-	15
	0.005	-	10	8	-	12
**3c**	0.010	8	8	10	8	14
	0.005	-	-	9	-	10
**3d**	0.010	-	-	8	-	8
	0.005	^-^	-	-	-	-
**3e**	0.010	13	13	19	10	15
	0.005	13	-	14	9	9

^a ^A: *Staphylococcus aureus*; B: *Escherichia coli*; C: *Candida albicans*; D: local resistant *Pseudomonas aeruginosa*; E: reference *Pseudomonas aeruginosa* ATCC 27853. ^b ^Inhibition zone diameter measured in mm.

**Table 2:** Sensitivity of human pathogenic microbes to the new synthetic heterocyclic compounds using the filter paper disk method.

Compound	Conc. (g/mL)	Pathogens				
		A^a^	B	C	D	E
**Tobramycin**	0.010	16^b ^	14	24	-	16
	0.005	10	12	16	-	10
						
**2a**	0.010	10	13	14	-	9
	0.005	8	10	8	-	-
**2b**	0.010	13	14	15	-	8
	0.005	9	10	9	-	-
**2c**	0.010	-	9	13	-	9
	0.005	-	-	9	-	-
**2d**	0.010	8	9	9	-	9
	0.005	-	-	-	-	-
**3a**	0.010	8	13	14	9	15
	0.005	-	10	8	-	11
**3b**	0.010	10	11	12	-	16
	0.005	-	10	9	-	11
**3c**	0.010	8	8	11	8	15
	0.005	-	-	10	-	11
**3d**	0.010	8	-	9	-	9
	0.005	^-^	-	-	-	-
**3e**	0.010	15	14	21	13	18
	0.005	14	10	15	10	11

^a ^A: *Staphylococcus aureus*; B: *Escherichia coli*; C: *Candida albicans*; D: local resistant *Pseudomonas aeruginosa*; E: reference *Pseudomonas aeruginosa* ATCC 27853. ^b ^Inhibition zone diameter measured in mm.

As indicated from the present data, these synthetic heterocyclic compounds show potential as novel antimicrobial agents, compared with the reference antibiotic tobramycin. Most of these heterocyclic compounds are active against *Candida albicans*, a eukaryotic cell, so these synthetic heterocyclic compounds should be studied further in the context of antitumor or anticancer drugs (chemotherapeutic agents).

## Experimental

### General

The melting points (m.p.) were determined on an Electro Thermal digital melting point apparatus. The solvents were purified by standard procedures. Infrared (IR) spectra were recorded using a NICOLET 410 FT-IR spectrometer (in cm^-1^). The IR spectra of pure substances were measured as KBr-pellets. NMR spectra were recorded on a Jeol JNM-ECP400 FT-NMR system at 400 MHz (^1^H-NMR) and 100 MHz (^13^C-NMR), respectively. The data are reported in delta (δ) units relative to tetramethylsilane (TMS) used as internal reference. The mass spectra were measured on a Finnigan MAT95 instrument (5 kV Ionizing energy, field desorption). The elemental analyses were performed in the analytical laboratory of the Institute of Organic Chemistry of the University of Mainz, Germany. *o*-, *m*- and *p*-Diacetylbenzene, and *p*-toluenesulfonyl hydrazide were obtained from Aldrich and 1,3,5-triacetylbenzene, epichlorohydrin and dimethyl sulfoxide were obtained from Acros. Tobramycin was obtained from Arcomex.

### General procedure for the preparation of the tosylhydrazones **2a-d**

A mixture of *p*-toluenesulfonyl hydrazide (0.60 mmol) and an equivalent amount of the appropriate ketones (*o*-, *m*-, *p*-diacetylbenzene and 1,3,5-triacetylbenzene, 0.30 mmol and 0.20 mmol, respectively) in absolute ethanol (35 mL) was refluxed for 1.5 hour. The reaction was followed by TLC in chloroform until completion. The solution was cooled in an ice bath, concentrated to one third of its original volume. The colorless solid product thus precipitated was filtered by vacuum filtration, washed with several portions of cold ethanol and, when necessary, a recrystalization from ethanol was performed.

*(E,E)-1,1’-[1,2-Phenylene)bisethanone-bis(tosylhydrazone)* (**2a**). Colorless powder, obtained in 73% yield; m.p.147°C (decomposition); ^1^H-NMR (DMSO-d_6_): δ = 2.14 (s, 6H, CH_3_), 2.21 (s, 6H, CH_3_-toluene), 7.33 (d, *J* = 7.6 Hz, 4H, toluene), 7.45 (d, *J* = 7.6 Hz, 4H, toluene), 7.57 (d, *J* = 7.4 Hz, 2H, phenyl), 7.68 (d, *J* = 7.4 Hz, 2H, phenyl), 10.11 (s, 2H, NH) ppm; ^13^C-NMR (DMSO-d_6_): δ = 14.6/20.9 (4C, CH_3_), 123.8 (2C, CH-phenyl), 126.2 (2C, CH-phenyl), 128.4 (2C, CC-phenyl), 148.5 (2C, CN), 128.1 (4C, CH-toluene), 129.6 (4C, CH-toluene), 136.4 (2C, CC-Toluene), 142.7 (2C, CS-toluene) ppm; IR (cm^-1^): ν = 3187, 1601, 1493, 1310, 1226, 921, 829; MS: m/z (%) 498 (100%); Anal. Calcd. for C_24_H_26_N_4_O_4_S_2_: C, 57.81; H, 5.26; N, 11.24; S, 12.86. Found: C, 57.35; H, 5.56; N, 11.09.

*(E,E)-1,1’-[1,3-Phenylene)bisethanone-bis(tosylhydrazone)* (**2b**). Colorless powder, obtained in 82% yield; m.p.163°C (decomposition); ^1^H-NMR (DMSO-d_6_): δ = 2.17 (s, 6H, CH_3_), 2.29 (s, 6H, CH_3_-toluene), 7.37 (d, *J* = 7.6 Hz, 4H, toluene), 7.46 (d, *J* = 7.6 Hz, 4H, toluene), 7.53 (t, *J* = 7.4 Hz, 1H, phenyl), 7.69 (d, J = 7.4 Hz, 2H, phenyl), 8.05 (s, 1H, phenyl), 10.14 (s, 2H, NH) ppm; ^13^C-NMR (DMSO-d_6_): δ = 14.5/20.4 (4C, CH_3_), 123.8 (1C, CH-phenyl), 126.9 (2C, CH-phenyl), 128.6 (2C, CC-phenyl), 138.3 (1C, CH-phenyl), 149.5 (2C, CN), 127.7 (4C, CH-toluene), 129.1 (4C, CH-toluene), 136.3 (2C, CC-toluene), 142.9 (2C, CS-toluene) ppm; IR (cm^-1^): ν = 3193, 1591, 1501, 1312, 1232, 910, 828; MS: m/z (%) 498 (100%); Anal. Calcd. for C_24_H_26_N_4_O_4_S_2_: C, 57.81; H, 5.26; N, 11.24; S, 12.86. Found: C, 57.42; H, 5.13; N, 11.13.

*(E,E)-1,1’-[1,4-Phenylene)bisethanone-bis(tosylhydrazone)* (**2c**). Colorless powder, obtained in 88% yield; m.p.186°C (decomposition); **1**H-NMR (DMSO-d_6_): δ = 2.20 (s, 6H, CH_3_), 2.32 (s, 6H, CH_3_-toluene), 7.39 (d, *J* = 7.6 Hz, 4H, toluene), 7.48 (d, *J* = 7.6 Hz, 4H, toluene), 7.75 (s, 4H, phenyl), 10.17 (s, 2H, NH) ppm; ^13^C-NMR (DMSO-d_6_): δ = 14.3/20.7 (4C, CH_3_), 126.3 (4C, CH-phenyl), 129.6 (2C, CC-phenyl), 148.9 (2C, CN), 128.1 (4C, CH-toluene), 129.3 (4C, CH-toluene), 136.7 (2C, CC-toluene), 143.1 (2C, CS-toluene) ppm; IR (cm^-1^): ν = 3213, 1591, 1521, 1312, 1246, 917, 826; MS: m/z (%) 498 (100%); Anal. Calcd. for C_24_H_26_N_4_O_4_S_2_: C, 57.81; H, 5.26; N, 11.24; S, 12.86. Found: C, 57.53; H, 5.44; N, 11.42.

*(E,E,E)-1,1’,1’’-[1,3,5-benzenetriyl)trisethanone-tris(tosylhydrazone)* (**2d**). Colorless powder, obtained in 84% yield; m.p.121°C (decomposition); ^1^H-NMR (DMSO-d_6_): δ = 2.23 (s, 9H, CH_3_), 2.35 (s, 9H, CH_3_-toluene), 7.41 (d, *J* = 7.6 Hz, 6H, toluene), 7.48 (d, *J* = 7.6 Hz, 6H, toluene), 8.01 (s, 3H, phenyl), 10.16 (s, 3H, NH) ppm; ^13^C-NMR (DMSO-d_6_): δ = 14.5/20.6 (6C, CH_3_), 124.4 (3C, CH-phenyl), 138.2 (3C, CC-phenyl), 148.6 (3C, CN), 128.8 (6C, CH-toluene), 129.6 (6C, CH-toluene), 136.5 (3C, CC-toluene), 142.7 (3C, CS-toluene) ppm; IR (cm^-1^): ν = 3189, 1587, 1584, 1506, 1321, 1243, 922, 825; MS: m/z (%) 708 (100%); Anal. Calcd. for C_33_H_36_N_6_O_6_S_3_: C, 55.92; H, 5.12; N, 11.86; S, 13.57. Found: C, 55.67; H, 5.24; N, 11.56.

### General procedure for the preparation of the 1,2,3-selenadiazole derivatives **3a-d** [16, 22]

Tosylhydrazone **2a-c** or **2d** (0.36 mmol) was mixed with an equivalent amount of selenium dioxide powder (0.72 mmol) or (1.08 mmol), respectively, and sodium sulfate (3.0 g) in 1,4-dioxane (20 mL) under vigorous stirring and gentle heating at 35-45°C. The solution was kept in the dark and the reaction progress was monitored by TLC, showing that the reactions complete within about 20 hours. After reducing in vacuo the volume of the reaction mixture to two thirds of its original volume, distilled water (30 mL) was added, and the ensuing mixture was extracted with chloroform (3x20 mL).. The combined chloroform layers were dried over sodium sulphate. The crude product was obtained after solvent removal in vacuo. Column chromatography using toluene/diethyl ether (7:3) as eluent was used to separate the product, which can be recrystallized subsequently from chloroform/hexane.

*1,2-Bis(1,2,3-selenadiazole-4-yl)benzene* (**3a**). Dark brown solid, obtained in 65% yield; m.p. 112°C (decomposition); ^1^H-NMR (CDCl_3_): δ = 7.75 (d, *J* = 7.4 Hz, 2H, phenyl), 8.23 (d, *J* = 7.4 Hz, 2H, phenyl), 8.97 (s, 2H, CHSe) ppm; ^13^C-NMR (CDCl_3_): δ = 124.7 (2C, CH-phenyl), 128.3 (2C, CH-phenyl), 129.6 (2C, CC-phenyl), 133.2 (2C, C-Se), 162.3 (2C, C-N) ppm; IR (cm^-1^): ν = 3053, 1632, 1481, 1406, 1269, 1216, 957; MS: m/z (%) 340 (100%); Anal. Calcd. For C_10_H_6_N_4_Se_2_: C, 35.32; H, 1.78; N, 16.47; Se, 46.44. Found: C, 35.27; H, 1.75; N, 16.41.

*1,3-Bis(1,2,3-selenadiazole-4-yl)benzene* (**3b**). Dark brown solid, obtained in 72% yield; m.p. 133°C (decomposition); ^1^H-NMR (CDCl_3_): δ = 7.69 (t, *J* = 7.4 Hz, 1H, phenyl), 8.15 (d, *J* = 7.4 Hz, 2H, phenyl), 8.86 (s, 1H, phenyl), 8.93 (s, 2H, CHSe) ppm; ^13^C-NMR (CDCl_3_): δ = 121.3 (1C, CH-phenyl), 124.3 (2C, CH-phenyl), 127.6 (1C, CH-phenyl), 130.1 (2C, CC-phenyl), 133.2 (2C, C-Se), 160.5 (2C, C-N) ppm; IR (cm^-1^): ν = 3060, 1628, 1476, 1419, 1253, 1225, 963; MS: m/z (%) 340 (100%); Anal. Calcd. For C_10_H_6_N_4_Se_2_: C, 35.32; H, 1.78; N, 16.47; Se, 46.44. Found: C, 35.27; H, 1.75; N, 16.41.

*1,4-Bis(1,2,3-selenadiazole-4-yl)benzene* (**3c**). Light brown solid, obtained in 78% yield; m.p. 122°C (decomposition); ^1^H-NMR (CDCl_3_): δ = 8.16 (s, 4H, CH-phenyl), 8.69 (s, 2H, CHSe) ppm; ^13^C-NMR (CDCl_3_): δ = 123.6 (4C, CH-phenyl), 129.4 (2C, CC-phenyl), 136.2 (2C, C-Se), 161.8 (2C, C-N) ppm; IR (cm^-1^): ν = 3068, 1635, 1490, 1410, 1260, 1220, 960; MS: m/z (%) 340 (100%); Anal. Calcd. For C_10_H_6_N_4_Se_2_: C, 35.32; H, 1.78; N, 16.47; Se, 46.44. Found: C, 35.29; H, 1.73; N, 16.43.

*1,3,5-Tris(1,2,3-selenadiazole-4-yl)benzene* (**3d**). Light brown solid, obtained in 81% yield; m.p. 131°C (decomposition); at 136°C the decomposed solid changed to a reddish brown liquid, while gas evolution occurred; ^1^H-NMR (CDCl_3_): δ = 8.95 (s, 3H, CH-phenyl), 9.18 (s, 3H, CHSe) ppm; ^13^C- NMR (CDCl_3_): δ = 122.9 (3C, CH-phenyl), 131.3 (3C, CC-phenyl), 138.8 (3C, C-Se), 162.1 (3C, C-N) ppm; IR (cm^-1^): ν = 3086, 1598, 1496, 1414, 1223, 978; MS: m/z (%) 471 (100%); Anal. Calcd. For C_12_H_6_N_6_Se_3_: C, 30.59; H, 1.28; N, 17.84; Se, 50.29. Found: C, 30.53; H, 1.25; N, 17.79.

### 3-[4-(1,2,3-Selenadiazole-4-yl)phenoxy]-1,2-propenoxide (**3e**)

This compound was obtained by reacting 4-(1,2,3-selenadiazole-4-yl)phenol (1.00 mmol) [20] with epichlorohydrin (2.00 mmol), potassium carbonate (2.00 mmol) and potassium iodide (2.00 mmol) in dry acetone (25 mL) under reflux. The reaction progress was followed by TLC (chloroform) showing the completion of the reaction after 20 hours. Then water (25 mL) was added to the mixture. The mixture was stirred for 5 min. and extracted with dichloromethane (3x15 mL). The organic phase was dried over magnesium sulfate and concentrated to dryness. The residue was separated by column chromatography on silica gel, using ethanol/dichloromethane (1:20) as eluant. The compound was obtained as a light brown solid in 71% yield; m.p.163°C; ^1^H-NMR (CDCl_3_): δ = 2.65 (q, ^3^*J*_cis_ = 4.2 Hz, 1H, 3'-H), 2.94 (t, ^3^*J*_trans_ = 2.3 Hz, 1H, 3'-H), 3.42 (m, ^3^*J*_cis_ = 4.5 Hz, 1H, 2'-H), 3.92 (q, ^3^*J*_trans_ = 2.2 Hz, 1H, 1'-H), 4.26 (q, ^3^*J*_cis_ = 4.3 Hz, 1H, 1'-H), 7.11 (d, *J* = 7.4 Hz, 2H, CH-phenyl), 7.89 (d, J = 7.4 Hz, 2H, CH-phenyl), 9.08 (s, 1H, CHSe) ppm; ^13^C-NMR (CDCl_3_): δ = 44.7 (1C, CH_2_-epoxide), 50.8 (1C, CH-epoxide), 69.8 (1C, CH_2_O), 115.7 (2C, CH-phenyl), 125.7 (1C, CC-phenyl), 128.9 (2C, CH-phenyl), 132.3 (1C, CHSe), 159.2 (1C, CO-phenyl), 162.2 (1C, CCSe) ppm; IR (cm^-1^): ν = 3088, 1602, 1523, 1462, 1253, 973; MS: m/z (%) 281 (100%); Anal. Calcd. For C_11_H_10_N_2_O_2_Se: C, 46.99; H, 3.56; N, 9.96; Se, 28.08. Found: C, 46.93; H, 3.43; N, 9.89.

### Microbiology

The filter paper disk and the hole diffusion methods were used to measure the inhibitory activity as indicated by the diameter of the inhibition zone [21,22]. The filter paper disk method was carried out as follows: Whatman III filter paper puncture disks 5 mm were saturated with different concentrations of the heterocyclic compounds to be tested. They were dried in an oven at 60°C for 12 hours. The dried disks were placed on already cultured nutrient agar plates concomitantly with the pathogens. The diameter of the clear zone around the disks was measured after 48 hours of incubation at 37°C. The absence of a clear zone around the disks indicated inactivity.

The hole method was carried out as follows: for each concentration, 100 µL of the heterocyclic compounds were placed in 5 mm diameter wells on nutrient agar inoculated spontaneously with the pathogens to be tested against. The plates were incubated at 37°C for 48 hours. The clear zone around the wells was measured as inhibition zones. The absence of a clear zone around the well was taken as inactivity. In both methods, DMSO was used as a solvent to prepare the solutions of the heterocyclic compounds.
